# Right ventricular dysfunction is a predictor of non-response and clinical outcome following cardiac resynchronization therapy

**DOI:** 10.1186/1532-429X-13-68

**Published:** 2011-10-31

**Authors:** Francisco Alpendurada, Kaushik Guha, Rakesh Sharma, Tevfik F Ismail, Amy Clifford, Winston Banya, Raad H Mohiaddin, Dudley J Pennell, Martin R Cowie, Theresa McDonagh, Sanjay K Prasad

**Affiliations:** 1 CMR Unit. Royal Brompton Hospital. Sydney Street. London, SW3 6NP. UK; 2 Department of Cardiology, Royal Brompton Hospital, Sydney Street, Chelsea, London, SW3 6NP, UK; 3 Research & Development, Royal Brompton Hospital, Sydney Street, Chelsea, London, SW3 6NP, UK; 4 Department of Cardiology. King's College Hospital, Denmark Hill, London, SE5 9RS, UK

**Keywords:** heart failure, cardiac resynchronization therapy, right ventricular function, cardiovascular magnetic resonance

## Abstract

**Background:**

Cardiac resynchronization therapy (CRT) is an established treatment in advanced heart failure (HF). However, an important subset does not derive a significant benefit. Despite an established predictive role in HF, the significance of right ventricular (RV) dysfunction in predicting clinical benefit from CRT remains unclear. We investigated the role of RV function, assessed by cardiovascular magnetic resonance (CMR), in predicting response to and major adverse clinical events in HF patients undergoing CRT.

**Methods:**

Sixty consecutive patients were evaluated with CMR prior to CRT implantation in a tertiary cardiac centre. The primary end-point was a composite of death from any cause or unplanned hospitalization for a major cardiovascular event. The secondary end-point was response to therapy, defined as improvement in left ventricular ejection fraction ≥ 5% on echocardiography at one year.

**Results:**

Eighteen patients (30%) met the primary end-point over a median follow-up period of 26 months, and 27 out of 56 patients (48%) were considered responders to CRT. On time-to-event analysis, only atrial fibrillation (HR 2.6, 95% CI 1.02-6.84, p = 0.047) and RV dysfunction, either by a reduced right ventricular ejection fraction-RVEF (HR 0.96, 95% CI 0.94-0.99, p = 0.006) or tricuspid annular plane systolic excursion-TAPSE (HR 0.88, 95% CI, 0.80-0.96, p = 0.006), were significant predictors of adverse events. On logistic regression analysis, preserved RVEF (OR 1.05, 95% CI 1.01-1.09, p = 0.01) and myocardial scar burden (OR 0.90, 95% CI 0.83-0.96, p = 0.004) were the sole independent predictors of response to CRT. Patients with marked RV dysfunction (RVEF < 30%) had a particularly low response rate (18.2%) to CRT.

**Conclusions:**

Right ventricular function is an important predictor of both response to CRT and long-term clinical outcome. Routine assessment of the right ventricle should be considered in the evaluation of patients for CRT.

## Background

Cardiac resynchronisation therapy (CRT) is an established therapeutic option for selected patients with symptomatic heart failure (HF). Amongst its benefits are reduced mortality, improved exercise tolerance and quality of life [1,2]. However, a proportion of patients do not gain any significant benefit, the reasons for which are unclear. Thus a number of devices are being implanted with no discernible clinical benefit, which has important healthcare costs implications, as well as exposing patients to unnecessary risks. Our current strategy for assessing benefit with CRT is mainly focused on assessing symptomatic or functional response, but it is increasingly clear that this does not necessarily translate into improved clinical outcomes. It is therefore important to refine the selection criteria for device implantation to better identify those who would benefit-both in terms of response and improved clinical outcomes.

Whilst much attention has focused on remodelling of the left ventricle (LV), the role of the right ventricle (RV) in the appropriate selection of patients for CRT remains unclear [3]. Previous studies assessing RV function have utilised echocardiography and radionuclide imaging [4-7]. However, accuracy of RV volumes and function by these techniques may be inaccurate due to the anatomical location and complex geometric structure. Cardiovascular magnetic resonance (CMR) offers superior three dimensional representation of the RV, leading to a more accurate and reproducible assessment of RV function [8]. We therefore sought to assess the impact of RV function on outcomes in HF patients undergoing CRT implantation using CMR.

## Methods

### Study population

We studied 60 consecutive patients attending the Royal Brompton Hospital heart failure pacing clinic between January 2005 and March 2010 who fulfilled the following criteria: 1. New York Heart Association (NYHA) class III/IV at the time of CRT implantation; 2. QRS width ≥ 120 ms; 3. LVEF ≤ 35% by echocardiography, and; 4. CMR study within 3 months before CRT implantation.

These patients were evaluated for clinical (aetiology of heart failure, symptom status and medication, heart rate, blood pressure) and electrocardiographic (rhythm and QRS width) parameters at the time of device implantation. As this study involved review of local patient medical records, individual consent was not required by our Ethics Committee who approved the study.

### Imaging

Cardiovascular magnetic resonance studies were performed in 1.5T Sonata or Avanto scanners (Siemens, Erlangen, Germany). A short-axis stack from atrio-ventricular level to the apex was acquired using a steady-state free-precession cine sequence (echo time 1.6 ms, repetition time 3.2 ms, flip angle 60°, slice thickness 7 mm with a 3 mm gap, acquisition time of 8-12 cardiac cycles) to quantify left and right ventricular volumes. Long-axis cines were also acquired to define the valve plane throughout the cardiac cycle. An inversion recovery gradient echo sequence was used 10 minutes after gadolinium injection (Magnevist^® ^or Gadovist^® ^, 0.1 mmol/kg) to assess myocardial scar. Inversion times were set to null the normal myocardium with images repeated in two stacks of identical short-axis planes but separate phase-encoding directions to exclude artefact.

**Figure 1:**
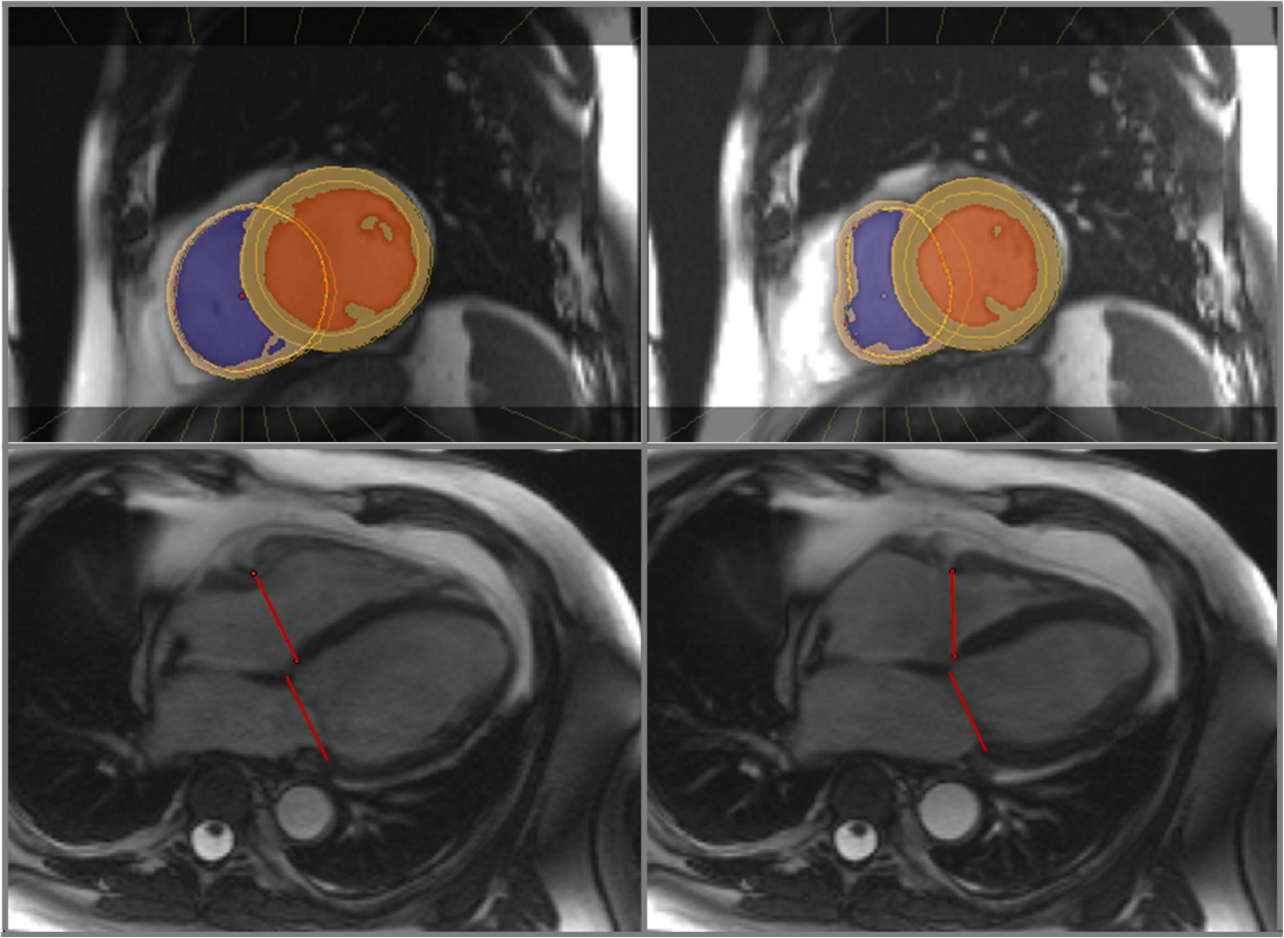
**Software used for ventricular volumes and mass measurements**. Mid-ventricular short-axis (top) and four-chamber views (bottom) at end-diastole (left) and end-systole (right). Coloured areas represent the left and right ventricular cavities and myocardium. Ventricular volumes are generated from a short-axis stack after being confined by the mitral and tricuspid valve planes (red lines).

**Figure 2:**
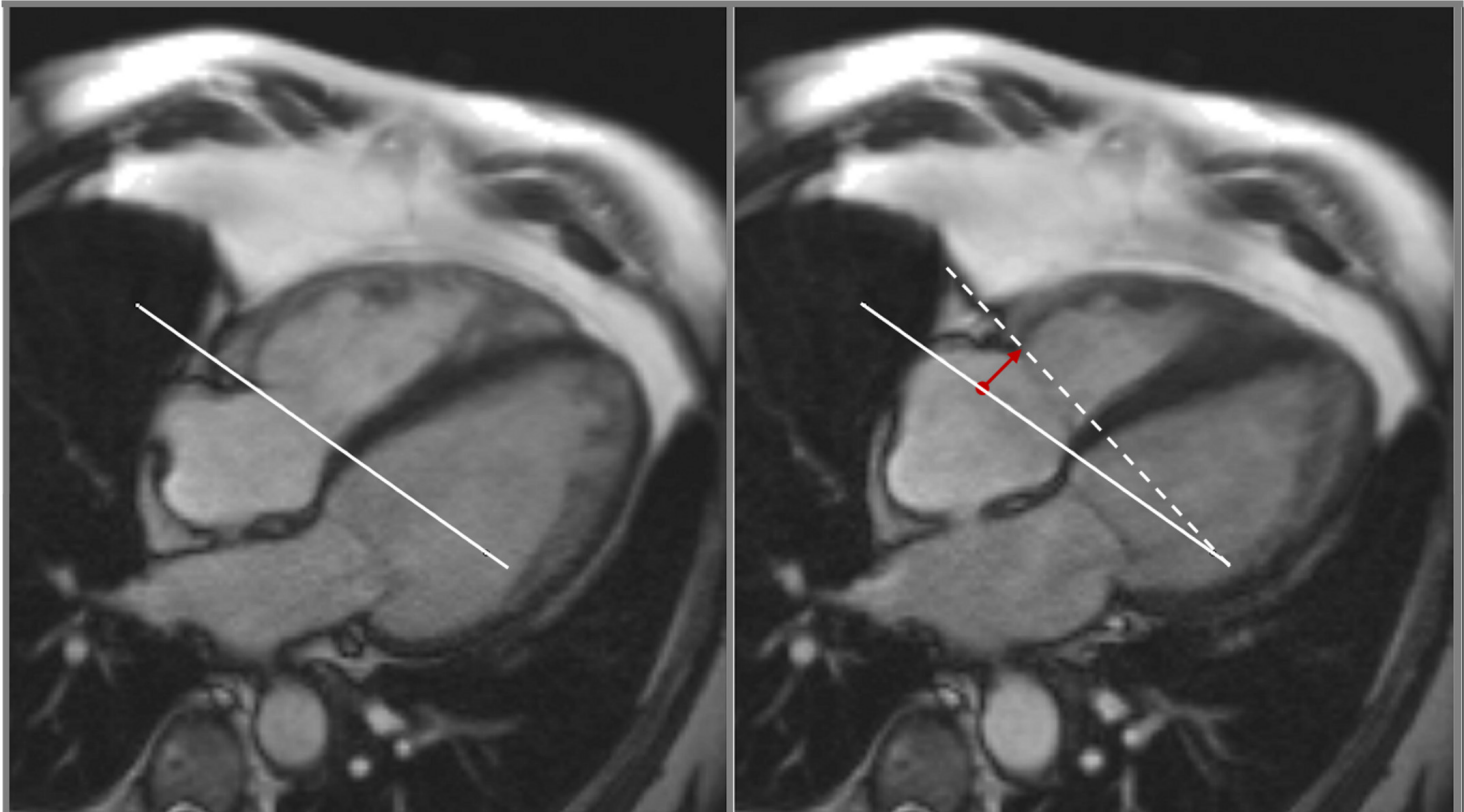
**Four-chamber view in end-diastole (left panel) and end-systole (right panel)**. The tricuspid annular plane is marked as a white line in diastole and as a dashed line in systole. The red vector represents the tricuspid annular systolic excursion (TAPSE).

Left and right ventricular volumes were calculated using semi-automated software (CMR tools, Cardiovascular Imaging Solutions, London, UK), as previously described (Figure 1) [9,10]. The resulting values were then indexed to body surface area and compared to reference values from a control population [9,10]. Tricuspid annular plane systolic excursion (TAPSE) was measured from the 4-chamber view (Figure 2). RV dysfunction was defined as RVEF < 50% or TAPSE < 15 mm; severe RV dysfunction was defined as RVEF < 30% or TAPSE < 10 mm. Peak RV wall thickness was measured from the short-axis slices.

Valvular regurgitation was graded as mild (n = 1), moderate (n = 2) or severe (n = 3) by blinded observers, based on the echocardiographic and CMR findings. LVEF was also calculated by echocardiography before and 1 year after device implantation using the Simpson's method from the 2-chamber and 4-chamber views. Pulmonary artery systolic pressure was determined by echocardiography using standard methodology.

Contrast imaging with gadolinium was performed to assess the aetiology of the heart failure. Assessment of late gadolinium enhancement (LGE) in the left or right ventricle was interpreted by blinded observers. When present, the amount of LGE was quantified in a 16-segment model based on the "full width at half maximum" technique by customized analysis software (MRI-MASS, Medis, Leiden, the Netherlands) [11].

### Outcomes

All patients were followed-up in a heart failure clinic and standard medications were adjusted and optimized at these appointments. Data was collected from local hospital records, nation-wide summary case records and the Office of National Statistics. The primary end-point was a composite of all-cause mortality or an unplanned hospitalization for a major cardiovascular event; only the first event in each patient was included in this analysis [2]. The secondary end-point was echocardiographic response to CRT, defined as an improvement in LVEF by more than 5% by echocardiography 12 months after CRT implantation [12,13].

### Statistical analysis

Categorical variables are presented as frequency and percentage. Continuous variables are presented as mean ± standard deviation (SD) if normally distributed, or as median plus inter-quartile range (IQR) as appropriate. Continuous variables were compared with Student's t-test or the Mann-Whitney for non-parametric data. Correlation was assessed with Pearson's or Spearman's methods as appropriate.

Time to first event analysis was performed using Cox's proportional hazard models. Log-rank test was used to compare Kaplan-Meier cumulative event curves. Logistic regression was used to assess response to CRT at 12 months. Multivariate logistic regression analysis using a forward stepwise approach was performed on parameters that were significant on univariate analysis.

A two-tailed value of p-value < 0.05 was considered significant. All statistical analysis was performed using SPSS 19.0 (IBM, Chicago, Illinois, USA).

## Results

### Patients

Patient baseline characteristics are presented in table 1. The mean age of the study population was 65.3 ± 12.5 years; most of the patients were male (76.7%). Heart failure was ischaemic in 48.3% of patients, and atrial fibrillation/flutter was found in 23.3% of patients. The mean heart rate was 76 ± 15 bpm, and the mean QRS interval was 156 ± 21 ms (LBBB morphology in 95.0%). The median time between CMR and device implantation was 6 days. Fifty-six out of the 60 CRT (93%) devices were combined with a defibrillator (CRT-D).

**Table 1:** Baseline characteristics of the patients

Age, years	65.3 ± 12.5
Male gender	46 (76.7%)

Heart failure, aetiology	
Dilated cardiomyopathy	27 (45.0%)
Ischaemic	29 (48.3%)
Valvular	3 (5.0%)
Congenital	1 (1.7%)

Rhythm	
Sinus	46 (76.7%)
Atrial fibrillation	13 (21.7%)
Atrial flutter	1 (1.7%)

Medication	
Beta-blockers	43 (71.7%)
ACE inhibitors/ARB	58 (96.7%)
Aldosterone antagonists	39 (65.0%)
Loop diuretics	52 (86.7%)
Digoxin	12 (20.0%)
Aspirin	23 (38.3%)
Warfarin	19 (31.7%)
Statin	30 (50.0%)

ECG	
QRS width (ms)	156 ± 21

Heart rate	
Beats per minute (bpm)	76 ± 15

Blood pressure	
Systolic (mmHg)	120 ± 20
Diastolic (mmHg)	72 ± 13

Left ventricle	
EDV (mL/m^2^)	169 ± 62
ESV (mL/m^2^)	124 ± 55
EF (%)	27 ± 8
Mass (g/m^2^)	113 ± 30

Right ventricle	
EDV (mL/m^2^)	82 (65-123)
ESV (mL/m^2^)	38 (27-76)
EF (%)	52 (37-62)
TAPSE (mm)	13.5 ± 5.6
Peak wall thickness (mm)	3.6 ± 0.9

Pulmonary artery pressure (mmHg)	38.7 ± 8.7

Late gadolinium enhancement	
Absent	20 (33.3%)
Subendocardial	19 (31.7%)
Subendocardial + mid-wall	6 (10.0%)
Mid-wall	15 (25.0%)
Right ventricle	5 (8.3%)

Data is presented as n (%), mean ± SD, or median (25^th ^- 75^th ^percentile). NYHA = New York Heart Association; ACE = angiotensin-converting enzyme; ARB = angiotensin receptor blocker; BNP = B-type natriuretic peptide; EDV = End-diastolic volume; ESV = End-systolic volume; EF = Ejection fraction; TAPSE = tricuspid annular plane systolic excursion.

### Left ventricle

Both mean left ventricular indexed end-diastolic and end-systolic volumes were high (169 ± 62 and 124 ± 55 mL/m^2^, respectively). The indexed left ventricular end-diastolic volume was increased in 93% of the patients, while the indexed left ventricular end-systolic volume was increased in 99% of the patients compared to normal reference values [9]. The mean LVEF was 27 ± 8%, and the mean mass index was increased at 113 ± 30 g/m^2^.

Mitral regurgitation was seen in 90% of the patients, and was significant in 25% of patients (18% moderate, 7% severe). Aortic regurgitation was seen in 25% of patients, and was moderate in only one patient.

### Right ventricle

Right ventricular indexed volumes and ejection fraction were non-normally distributed, and hence presented as median plus inter-quartile ranges. The median end-diastolic and end-systolic volumes were 82 (65-123) mL/m^2 ^and 38 (27-76) mL/m^2^, respectively. Right ventricular indexed end-diastolic volume was increased in 38% of patients, while the indexed end-systolic volume was increased in 53% of patients [10]. The median RVEF was 52% (IQR 37-62%), with 53% of patients having a RVEF ≥ 50%.

The mean TAPSE was 13.5 ± 5.6 mm, with 40% of patients having values within normal limits when the standard echocardiographic cut-off value of 15 mm was considered. The mean peak RV wall thickness was 3.6 ± 0.9 mm.

In the 52 patients (87%) in whom it could be measured, the mean PASP was 38.7 ± 8.7 mmHg, with 37% of patients having a PAP > 40 mmHg. Tricuspid regurgitation was seen in 57% of the patients, and was significant in 14% of patients (12% moderate, 2% severe). No significant pulmonary regurgitation was identified in any patient.

There was a moderately strong correlation between RVEF with TAPSE (r = 0.58, p < 0.01), and a moderate correlation between RVEF and LVEF (r = 0.41, p < 0.01). Severity of mitral regurgitation was associated with a higher PAP (p = 0.01) and a lower RVEF (p < 0.01). Modest inverse correlations were observed between RVEF and pulmonary artery pressure (r = -0.37, p < 0.01), RV wall thickness (r = -0.28, p = 0.03) and severity of tricuspid regurgitation (p = 0.03). TAPSE was inversely related with RV wall thickness (r = -0.29, p = 0.03), but not with pulmonary artery pressure (r = -0.15, p = 0.28) or LVEF (r = 0.09, p = 0.48).

RVEF was similar in patients with an ischaemic and non-ischaemic aetiology (46.9 ± 14.8% vs. 49.5 ± 18.9, p = 0.56), and in patients with AF compared to those in sinus rhythm (45.4 ± 14.9% vs. 49.2 ± 17.8%, p = 0.48).

### Myocardial fibrosis

Left ventricular LGE was present in two-thirds of the patients: 19 patients (31.7%) had sub-endocardial enhancement suggesting myocardial infarction, 15 patients (25.0%) had a mid-wall enhancement pattern indicating fibrosis, and the remaining 6 patients (10.0%) had a mixed pattern of myocardial infarction and mid-wall fibrosis. Myocardial infarction was therefore present in 25 patients (41.7%): the septal wall was affected in 11 patients; the inferolateral wall was involved in 15 patients. The median percentage of scar in the myocardium was 4% (IQR 0-18%).

Right ventricular LGE was present in 5 patients (8.3%). All these patients had coronary artery disease with inferior myocardial infarctions of the LV extending to the inferior free wall of the RV.

### Follow-up

**Figure 3:**
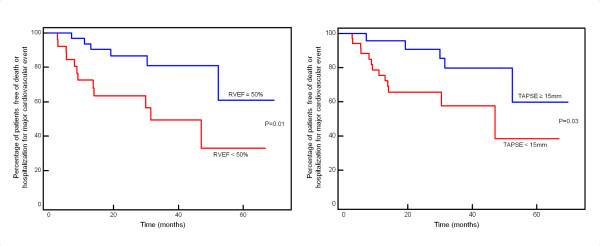
**Kaplan-Meier estimates of the time to the primary end-point for RVEF (left panel) and TAPSE (right panel)**.

During a median follow-up of 26.1 months (IQR 16.1-39.3 months) after CRT implantation, there were 13 unplanned hospitalizations for a cardiovascular cause (all for heart failure decompensation), and 11 deaths (8 cardiac and 3 non-cardiac deaths). The primary end-point was reached by 18 patients. Atrial fibrillation and RV dysfunction emerged as the only predictors of the primary composite end-point on univariate analysis (table 2). Patients in atrial fibrillation or flutter had a HR of 2.6 (95% CI 1.02-6.84, p = 0.047) for the primary end-point. For each 10% decrease in RVEF, the risk of the primary end-point increased by 40% (HR 0.96, 95% CI 0.94-0.99, p = 0.006); for each 1 mm decrease in TAPSE, the risk of the primary end-point increased by 12% (HR 0.88, 95% CI 0.80-0.96, p = 0.006). Kaplan-Meier curves for RV function assessed by RVEF or TAPSE are displayed on Figure 3.

**Table 2:** Univariate analysis: Primary end-point (time to death from any cause or an unplanned hospitalization for a major cardiovascular event).

	Events (n = 18)	No Events (n = 42)	HR	95% CI	P value
Age (years)	69.6 ± 11.9	63.3 ± 12.3	1.04	0.99-1.09	0.07
Male gender	14 (78%)	32 (76%)	1.14	0.37-3.46	0.82
CAD	10 (56%)	17 (41%)	1.73	0.68-4.38	0.25
Atrial fibrillation	6 (39%)	8 (17%)	2.63	1.02-6.84	0.047
Heart rate (bpm)	79.2 ± 19.4	74.1 ± 12.5	1.02	0.99-1.05	0.22
SBP (mmHg)	124 ± 22	118 ± 19	1.01	0.99-1.04	0.36
QRS width (ms)	151 ± 25	158 ± 21	0.99	0.96-1.01	0.23

Left ventricle					
EDV index (mL/m^2^)	159 ± 54	173 ± 64	0.99	0.98-1.00	0.09
ESV index (mL/m^2^)	120 ± 52	126 ± 56	0.99	0.98-1.00	0.19
EF (%)	26.9 ± 9.5	28.9 ± 7.6	1.00	0.94-1.06	0.93
Mass index (g/m^2^)	106 ± 25	115 ± 32	0.98	0.97-1.00	0.08
Mitral regurgitation	1.3 ± 0.6	1.2 ± 0.8	1.33	0.73-2.44	0.35
LGE	13 (72%)	27 (64%)	1.57	0.50-3.98	0.51
LGE (%)	9.5 (0-20)	3 (0-17)	1.01	0.98-1.05	0.54
Myocardial infarction	9 (50%)	16 (38%)	1.57	0.62-3.95	0.34

Right ventricle					
EDV index (mL/m^2^)	95 (76-138)	77 (60-103)	1.01	1.00-1.02	0.12
ESV index (mL/m^2^)	52 (35-102)	34 (21-66)	1.01	1.00-1.02	0.06
EF (%)	39 (25-54)	55 (43-66)	0.96	0.94-0.99	0.006
TAPSE (mm)	11.0 ± 4.4	14.5 ± 5.9	0.88	0.80-0.96	0.006
Wall thickness (mm)	3.9 ± 1.0	3.5 ± 0.9	1.54	0.93-2.56	0.09

PAP (mmHg)	42.5 ± 8.3	37.5 ± 8.5	1.06	1.00-1.12	0.07

Values are n (%), mean ± SD, or median (25^th ^- 75^th ^percentile). HR = hazard ratio; CI = confidence interval. CAD = Coronary artery disease; SBP = systolic blood pressure; LGE = late gadolinium enhancement; PAP = Pulmonary artery pressure. Other abbreviations as in table 1.

After documenting AF as a predictor of outcomes, we performed a post-hoc analysis on delivery rate, defined as the percentage of paced left ventricular beats. Data was collected from routine pacing clinic visits during the first year of implantation. Unlike AF, delivery rate was not associated with the primary end-point (HR 0.97, 95% CI 0.88-1.08, p = 0.56).

### Response to therapy

Of the 56 patients (93%) followed-up for over a year, 27 were considered to be responders as they had an increase in LVEF > 5% at one year of follow-up. Thus the response rate using this criterion was 48% (table 3).

**Table 3:** Univariate analysis: Response to cardiac resynchronization therapy (improvement of LVEF ≥ 5% at one year).

Response	Response (n = 27)	No Response (n = 29)	OR	95% CI	P value
Age	64.0 ± 12.2	65.8 ± 12.2	0.99	0.95-1.03	0.58
Male gender	18 (67%)	25 (86%)	0.32	0.09-1.20	0.09
CAD	7 (26%)	19 (66%)	0.18	0.06-0.58	0.004
Atrial fibrillation	4 (15%)	7 (24%)	0.55	0.14-2.13	0.38
Heart rate (bpm)	76 ± 15	76 ± 16	1.00	0.97-1.04	0.89
SBP (mmHg)	120 ± 20	119 ± 19	1.00	0.98-1.03	0.76
QRS width (ms)	161 ± 21	150 ± 22	1.02	1.00-1.05	0.07

Left ventricle					
EDV index (mL/m^2^)	156 ± 46	177 ± 71	0.99	0.98-1.00	0.19
ESV index (mL/m^2^)	111 ± 40	133 ± 63	0.99	0.98-1.00	0.13
EF (%)	30.5 ± 8.5	26.5 ± 7.2	1.07	0.99-1.16	0.07
Mass index (g/m^2^)	108 ± 26	118 ± 34	0.99	0.97-1.01	0.23
Mitral regurgitation	1.1 ± 0.6	1.3 ± 0.8	0.58	0.27-1.27	0.17
LGE	13 (48%)	24 (83%)	0.19	0.06-0.66	0.009
LGE (%)	0 (0-7)	13 (3-22)	0.91	0.85-0.97	0.005
Myocardial infarction	6 (22%)	18 (62%)	0.18	0.05-0.57	0.004

Right ventricle					
EDV index (mL/m^2^)	77 (67-104)	83 (61-139)	0.99	0.98-1.01	0.28
ESV index (mL/m^2^)	36 (23-54)	40 (31-96)	0.99	0.98-1.00	0.10
EF (%)	56 (45-63)	44 (28-61)	1.04	1.01-1.08	0.03
TAPSE (mm)	15.3 ± 5.8	11.8 ± 4.2	1.15	1.03-1.29	0.02
Wall thickness (mm)	3.3 ± 0.7	4.0 ± 1.0	0.42	0.21-0.84	0.02

PAP (mmHg)	36.9 ± 8.5	40.5 ± 9.0	0.95	0.89-1.02	0.16

Values are n (%), mean ± SD, or median (25th-75th percentile). OR = odds ratio; CI = confidence interval. Other abbreviations as in tables 1 and 2.

Two sets of closely related parameters were associated with non-response to CRT on univariate analysis: (1) coronary artery disease, associated with myocardial infarction and scar burden (myocardial fibrosis plus infarction relative to LV mass), were predictors of non-response to therapy; (2) right ventricular dysfunction (lower RVEF and TAPSE) and hypertrophy (thicker RV free wall). Myocardial scar burden (OR 0.90, 95% CI 0.83-0.96, p = 0.004) and RVEF (OR 1.05, 95% CI 1.01-1.09, p = 0.01) were the only variables to remain significant on multivariate analysis (table 4).

**Table 4:** Multivariate analysis: Response to cardiac resynchronization therapy (improvement of LVEF ≥ 5% at one year).

Response	Response (n = 27)	No Response (n = 29)	OR	95% CI	P value
LGE (%)	0 (0-7)	13 (3-22)	0.90	0.83-0.96	0.004
RVEF (%)	56 (45-63)	44 (28-61)	1.05	1.01-1.09	0.01

Values are median (25th-75th percentile). OR = odds ratio; CI = confidence interval; LGE = late gadolinium enhancement; RVEF = right ventricular ejection fraction

**Figure 4:**
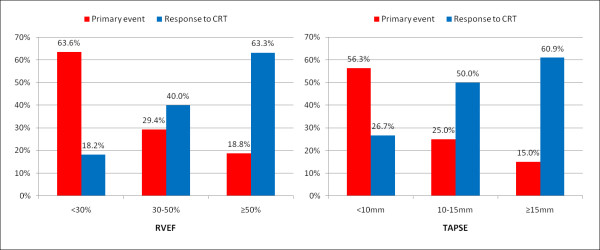
**Frequency of primary events and response to CRT for different ranges of RVEF and TAPSE**.

Response to CRT was noted to be lower as RV function deteriorated (Figure 4). Poor RV function, defined as a RVEF < 30% or a TAPSE < 10 mm, was associated with a particularly low response to CRT (response rates of 18.2% and 26.7%, respectively).

## Discussion

This study shows that RV dysfunction is associated with non-response and adverse outcomes in patients on CRT. Despite meeting the standard criteria for CRT implantation, there was a heterogeneous distribution of RV function in our study population. Since LVEF was narrowly distributed by accepted indications for CRT, the wide RVEF distribution observed may indeed explain why RV function was an independent discriminator in this population with advanced HF. A lower RVEF was associated with a lower LVEF, more significant mitral regurgitation, and a higher PAP. This suggests that the RV dysfunction may derive from two fundamental mechanisms, the importance of which may vary from patient to patient: 1) an impairment of global biventricular intrinsic contractility; 2) pulmonary hypertension secondary to elevated LV filling pressures and mitral regurgitation.

Adverse RV function may reflect more extensive and severe cardiac disease, to which improving myocardial synchrony has little impact. Of note, the response rate of the subgroup of patients with RVEF < 0.3 was less than 20%. Thus, patients with poor RV function were unlikely to benefit from CRT. This may have implications in stratifying patients for device therapy.

### Previous work

Few studies have investigated RV function and dyssynchrony. Previous work has shown that anything up to 20-40% of RV systolic pressure and volume load may originate from contraction from the LV [14]. Conversely, left bundle branch block (LBBB) may interfere with ventricular activation and induce mechanical dyssynchrony. Hence LBBB not only affects LV function but may impair RV function as well.

RV functional indices have been previously evaluated in CRT populations. Initial work by Field et al assessed RV function on adverse outcomes in a population of 77 patients undergoing CRT. This study showed that RV dysfunction measured by the Doppler-derived Tei index was a predictor of the composite end-point of death, transplantation and the implantation of left ventricular assist devices [4]. More recent work focused on RV function and response to CRT. In a study of 44 patients undergoing CRT, echocardiographic indices of RV function (TAPSE and RV fractional area change) were significantly worse in non-responders versus responders to CRT [5]. The value of TAPSE in predicting reverse remodelling was further validated in the CARE-HF population [6]. A further radionuclide imaging study in 44 patients undergoing CRT showed that those with a low baseline RVEF were less likely to improve in NYHA class, and tended to improve less in functional capacity and LVEF [7].

The results of our study are in line with the above publications. However, we used CMR as the gold-standard technique for assessment of the RV, and RVEF as the reference marker of RV performance. Along with myocardial infarction, RVEF was an independent predictor of response to CRT. Among an array of established prognostic markers in HF, RVEF and TAPSE emerged as the strongest predictors of events. Our findings correlated response with outcomes over a clinically relevant timeframe, thus supporting the potential of RV function as a predictor of short-term response as well as longer-term outcome following CRT.

### Tricuspid annular plane systolic excursion

Although available to all imaging modalities, and despite its validated prognostic value amongst cardiac and pulmonary conditions, RVEF estimation can be challenging as the right ventricle is a complex structure which cannot be modelled with simple geometric assumptions. Therefore, there is some interest in finding simpler ways for assessing RV performance. Of these, tricuspid annular plane systolic excursion (TAPSE) is the most described alternative to RVEF in the literature [15]. This marker of RV longitudinal function is reproducible and easy to obtain, and has shown to be a predictor of adverse outcomes in heart failure, irrespective of NYHA status and LV function [16,17]. In this study, TAPSE not only identified which patients would respond to CRT, but also the patients more likely to have major adverse events. Thus, TAPSE may be used by CMR as an alternative to RVEF for assessing RV function pre CRT when the latter cannot be estimated.

### Pulmonary hypertension

Increased pulmonary artery pressures have shown to be associated with non-response to CRT [5]. As ejection fraction is influenced by the afterload, it is expected for increasing pulmonary pressures to be associated with decreasing RVEF. A significant negative correlation was indeed observed between PAP and RVEF in our study. However, this correlation was modest and not as strong as other parameters such as LVEF, suggesting that factors other than pulmonary hypertension play a role in RV function. There was a trend for non-response to CRT and adverse outcomes in patients with increased pulmonary pressures, but statistical significance was not reached, probably explained by the smaller study population compared to other studies [18]. On the other hand, markers of RV function were significantly associated with response and adverse events. This suggests that RV function is more important than pulmonary pressure alone for both mechanical response and long-term prognosis in patients on CRT.

### Myocardial fibrosis

Late gadolinium enhancement CMR detects myocardial infarction accurately, with superior histological correlation compared to nuclear techniques [19]. This has become an active area of research in the CRT setting, with several subsequent studies documenting the value of scar burden, location and transmurality of myocardial infarctions as a predictor of response to CRT [20-24]. Consistent with this work, our study showed myocardial infarction and scar burden (as determined by percentage of LGE mass) to portend an adverse response to CRT. Although the amount of septal and lateral scar was associated with non-response to therapy (OR 0.99, 95% CI 0.98-1.00, p = 0.02; and OR 0.98, 95% CI 0.97-1.00, p = 0.02, respectively), scar location did not remain significant after including the total amount of scar in a multivariate model.

The lack of response observed with the above parameters did not translate into poorer outcomes. It is possible that this study was underpowered to detect more adverse events, as larger studies have shown ischaemic heart disease and location of myocardial infarction to portend a worse prognosis in CRT [25-27].

### Atrial fibrillation

Atrial fibrillation was the other predictor of outcomes besides RV dysfunction. This mirrors findings of long term studies of patients on CRT [28]. It is recognised that AF with a high ventricular rate may impair the delivery of biventricular pacing, and consequently undermine the clinical benefit of CRT [29]. Despite the paucity of data from randomized control trials in AF populations, recent guidelines support the use of CRT in AF patients, although restricted to a slightly wider QRS duration > 130 ms [30]. A recent European registry suggests that around 20% of patients undergoing CRT are actually in permanent AF, which is in keeping with our findings [31]. Of note, all but one AF patient in this study had QRS > 130 ms. Rate control was satisfactory in our AF cohort, the ventricular delivery rate was high (median 97%, IQR 91-99%), and none of the patients underwent AV nodal ablation. Unlike RV dysfunction, AF was not associated with a lower response rate, suggesting that the impact of AF on adverse outcomes was not altered by device implantation.

### Response

In addition to the hard clinical endpoints, we assessed the role of RV function on response to CRT. Response is a contentious topic, with various proposed definitions but no consensus amongst different criteria. The response rate varies widely, depending on the study population and the response criteria used. In a recent paper comparing 15 response criteria from the most cited papers, the response rate ranged from 32% to 91%. The same study showed that the agreement between different criteria was poor in 75% of the time, especially between clinical and echocardiographic criteria [32]. For our study, we used LVEF to assess response because it is an objective parameter and is also one of the selection criteria for CRT. In keeping with previous work, response for this study was an improvement in LVEF ≥ 5% at 12 months [12,13]. The response rate observed was relatively low (48%), but still in accordance with the available literature [32]. Using this criterion, RV dysfunction predicted a failure of response to CRT in terms of LV remodelling, thus supporting previous echocardiographic and radionuclide studies [5-7]. It is possible that significant RV dysfunction marks extensive and irreversible adverse remodelling, preventing reverse remodelling and functional recovery after CRT implantation. Improvement in LVEF ≥ 5% was significantly associated with event-free survival in this cohort of patients (likelihood ratio 4.7, p = 0.01). Mechanical remodelling thus appears to be a good surrogate marker of response to therapy, in line with a recently published analysis of the MADIT-CRT trial, where it was demonstrated that echocardiographic improvement in LV volumes and ejection fraction was associated with improved outcomes [33].

### Limitations

This was a retrospective study with a relatively small number of patients and events limiting multivariate analysis on outcomes. Nonetheless, both RVEF and TAPSE emerged as the most significant prognostic markers, suggesting that RV function as assessed by CMR has a powerful role in advanced HF undergoing CRT.

This was a single centre study in a tertiary referral hospital, but it allowed for consistent CMR scanning protocols, and consistent patient selection for CRT and close follow-up of all patients. To ensure a representative cohort, consecutive patients were identified.

The CRT devices implanted during this time period were non-MRI compatible and hence necessitated an alternative imaging modality for consequent follow-up. The development of CMR-compatible CRT devices could overcome this current limitation and may provide new insights on the response of both left and right ventricle to CRT.

## Conclusions

Right ventricular function appears an important predictor of response and major adverse events following CRT implantation. As current selection criteria only include patients with poor LV function, it is reasonable to assume that RV function will act as an important discriminative prognostic marker in patients undergoing CRT. Furthermore, poor RV function appears to identify a subgroup of patients with extensive ventricular remodelling unlikely to change their natural history. Therefore, CRT may be of no benefit in this subgroup of patients. The presence and amount of myocardial fibrosis, by contrast, was a predictor of response but did not predict outcomes.

The results of this study suggest that assessment of RV function can provide valuable information pre CRT implantation. Further work in larger cohorts is required to ascertain the precise role of RV evaluation in the selection of patients for CRT as well as the mechanisms for this dysfunction.

## List of abbreviations

CMR: cardiovascular magnetic resonance; CRT: cardiac resynchronization therapy; EF: ejection fraction; HF: heart failure; IQR: interquartile range; LBBB: left bundle brunch block; LV: left ventricle; PAP: pulmonary artery systolic pressure; RV: right ventricle; SD: standard deviation; TAPSE: tricuspid annular plane systolic excursion.

## Competing interests

KG has received support from Biotronik. RS has received honoraria/research grants from Medtronic, Boston Scientific, Biotronik and Servier. DJP is a consultant to Siemens, and a director in Cardiovascular Imaging Solutions. MRC has received research funding from Medtronic, St. Jude Medical and Boston Scientific.

## Authors' contributions

FA, KG, RS and SKP conceived the study. FA, KG, TFI and AC collected the data. WB performed the statistical analysis. All authors drafted, reviewed and approved the manuscript.
